# Correction: A chemical probe of CARM1 alters epigenetic plasticity against breast cancer cell invasion

**DOI:** 10.7554/eLife.63994

**Published:** 2020-10-15

**Authors:** Xiao-Chuan Cai, Tuo Zhang, Eui-jun Kim, Ming Jiang, Ke Wang, Junyi Wang, Shi Chen, Nawei Zhang, Hong Wu, Fengling Li, Carlo C dela Seña, Hong Zeng, Victor Vivcharuk, Xiang Niu, Weihong Zheng, Jonghan P Lee, Yuling Chen, Dalia Barsyte, Magda Szewczyk, Taraneh Hajian, Glorymar Ibáñez, Aiping Dong, Ludmila Dombrovski, Zhenyu Zhang, Haiteng Deng, Jinrong Min, Cheryl H Arrowsmith, Linas Mazutis, Lei Shi, Masoud Vedadi, Peter J Brown, Jenny Xiang, Li-Xuan Qin, Wei Xu, Minkui Luo

Cai X-C, Zhang T, Kim E-J, Jiang M, Wang K, Wang J, Chen S, Zhang N, Wu H, Li F, dela Seña CC, Zeng H, Vivcharuk V, Niu X, Zheng W, Lee JP, Chen Y, Barsyte D, Szewczyk M, Hajian T, Ibáñez G, Dong A, Dombrovski L, Zhang Z, Deng H, Min J, Arrowsmith CH, Mazutis L, Shi L, Vedadi M, Brown PJ, Xiang J, Qin L-X, Xu W, Luo M. 2019. A chemical probe of CARM1 alters epigenetic plasticity against breast cancer cell invasion. *eLife* **8**:e47110. doi: 10.7554/eLife.47110.

Published 28, October 2019

Recently, while revisiting the PDB deposition 6D2L, the structure of CARM1 in complex with 5a, we were noted that the beamline was incorrectly reported. An initial structure was solved using data collected at the Canadian Light Source. During the revision of the manuscript, we were able to obtain a higher resolution structure from beamline 24-ID-E (NE-CAT) at the Argonne National Library.

Below is the original text from the Materials and methods subsection “Crystallization of CARM1 in complex with 1 and 5a”:

“Graphics program COOTS4 was used for model building and visualization. Crystal diffraction data and refinement statistics for the structure are displayed in Table 2. To further confirm the electronic densities of the ligand, the total omission electron density map was calculated using SFCHECK from CCP4suite and contoured at 1.0 s (Figure 4—figure supplement 1) (Emsley and Cowtan, 2004; Murshudov et al., 1997).

For the CARM1-**5a** complex, CARM1 was crystallized with the sitting drop vapor diffusion method at 20°C by mixing 1 μL of protein solution with 1 μL of the reservoir solution containing 25% PEGG3350, 0.1M ammonium sulfate and 0.1 M Hepes (pH = 7.5). The compound **5a** (0.2 μL of 10 μM in DMSO) was added to the drops with apo crystals and incubated overnight. X-ray diffraction data for the CARM1-5a complex were collected at 100 K on a Rigaku FR-E superbright X-ray generator. Data were processed using the HKL-3000 suite (Minor et al., 2006). The structure was isomorphous to PDB entry 4IKP, which was used as a starting model. REFMAC (Murshudov et al., 2011) was used for structure refinement. Geometric restraints for compound refinement were prepared with GRADE v.1.102 developed at Global Phasing Ltd. (Cambridge, UK). The COOT graphics program (Emsley et al., 2010) was used for model building and visualization, and MOLPROBITY (Williams et al., 2018) was used for structure validation. To further confirm the electronic densities of the ligand, the total omission electron density map was calculated using SFCHECK from the CCP4suite and contoured at 1.0 s (Figure 3b) (Emsley and Cowtan, 2004; Murshudov et al., 1997).”

And the corrected version with changed text underlined:

“Graphics program COOT was used for model building and visualization. Crystal diffraction data and refinement statistics for the structure are displayed in Table 2. To further confirm the electronic densities of the ligand, the total omission electron density map was calculated using SFCHECK from CCP4suite and contoured at 1.0 sigma (Figure 4—figure supplement 1) (Emsley and Cowtan, 2004; Murshudov et al., 1997).

For the CARM1-**5a** complex, CARM1 was crystallized with the sitting drop vapor diffusion method at 20°C by mixing 1 μL of protein solution with 1 μL of the reservoir solution containing 25% PEGG3350, 0.1M ammonium sulfate and 0.1 M Hepes (pH = 7.5). The compound 5a (0.2 μL of 10 μM in DMSO) was added to the drops with apo crystals and incubated overnight. X-ray diffraction data for the CARM1-**5a** complex were collected at 100 K at beamline 24ID-E of Advanced Photon Source (APS), Argonne National Laboratory. Data were processed using the HKL-3000 suite (Minor et al., 2006). The structure was isomorphous to PDB entry 4IKP, which was used as a starting model. REFMAC (Murshudov et al., 1997) was used for structure refinement. Geometric restraints for compound refinement were prepared with GRADE v.1.102 developed at Global Phasing Ltd. (Cambridge, UK). The COOT graphics program (Emsley et al., 2010) was used for model building and visualization, and MOLPROBITY (Williams et al., 2018) was used for structure validation. To further confirm the electronic densities of the ligand, the total omission electron density map was calculated and contoured at 2.5 sigma (Figure 3b) (Emsley and Cowtan, 2004; Murshudov et al., 1997).”

Below is the original text from the Acknowledgements:

“The X-ray structure results for CARM1 are derived from work performed at Argonne National Laboratory, Structural Biology Center (SBC) at the Advanced Photon Source. SBC-CAT is operated by U Chicago Argonne, LLC, for the US Department of Energy, Office of Biological and Environmental Research under contract DE-AC02-06CH11357. These experiments were performed using beamline 08ID-1 at the Canadian Light Source, which is supported by the Canada Foundation for Innovation, Natural Sciences and Engineering Research Council of Canada, the University of Saskatchewan, the Government of Saskatchewan, Western Economic Diversification Canada, the National Research Council Canada, and the Canadian Institutes of Health Research.”

And the corrected version with changed text underlined:

“The X-ray structure results for CARM1 are derived from work conducted at the Northeastern Collaborative Access Team beamlines, which are funded by the National Institute of General Medical Sciences from the National Institutes of Health (P30 GM124165). The Eiger 16M detector on the 24-ID-E (NE-CAT) beam line is funded by a NIH-ORIP HEI grant (S10OD021527). This research used resources of the Advanced Photon Source, a U.S. Department of Energy (DOE) Office of Science User Facility operated for the DOE Office of Science by Argonne National Laboratory under Contract No. DE-AC02-06CH11357. Additional work was performed using beamline 23-ID-B (GM/CA). GM/CA@APS has been funded in whole or in part with Federal funds from the National Cancer Institute (ACB-12002) and the National Institute of General Medical Sciences (AGM-12006). The Eiger 16M detector at GM/CA-XSD was funded by NIH grant S10 OD012289.”

Below is the original text from the legend of Figure 3:

“The structure of SNF was extracted from a CARM1**–SNF–H3R17** complex (PDB 5D × 0). (**d**) Key interactions between CARM1 and ligands in canonical and noncanonical binding modes. The differentiated interactions are highlighted in gray (CARM1) and blue (**SNF**) for the canonical mode; and in green (CARM1) and orange (**5a**) for the noncanonical mode. (**e**) Additional interactions in which the α-amino amide moiety of **5a** forms hydrogen bonds with Glu266 and His414 and hydrophobic interactions with Phe152 and Tyr261. (**f**) Steric clash between the α-amino amide moiety of **5a** and an Arg substrate. The structure of the Arg substrate was extracted from a CARM1**–SNF–H3R17** complex (PDB 5D × 0).”

And the corrected version with changed text underlined:

“The structure of SNF was extracted from a CARM1**–SNF–H3R17** complex (PDB 5DX0). (**d**) Key interactions between CARM1 and ligands in canonical and noncanonical binding modes. The differentiated interactions are highlighted in gray (CARM1) and blue (**SNF**) for the canonical mode; and in green (CARM1) and orange (**5a**) for the noncanonical mode. (**e**) Additional interactions in which the α-amino amide moiety of **5a** forms hydrogen bonds with Glu266 and His414 and hydrophobic interactions with Phe152 and Tyr261. (**f**) Steric clash between the α-amino amide moiety of **5a** and an Arg substrate. The structure of the Arg substrate was extracted from a CARM1**–SNF–H3R17** complex (PDB 5DX0).”

The following reference was removed:

GN Murshudov, P Skubák, AA Lebedevk, NS Pannu, RA Steiner, RA Nicholls, MD Winn, F Long, AA Vagin. 2011. REFMAC5 for the refinement of macromolecular crystal structures. *Acta Crystallographica Section D Biological Crystallography* **67**:355–367. doi: https://doi.org/10.1107/S0907444911001314

